# Characteristics and co-admissions of mothers and babies admitted to residential parenting services in the year following birth in NSW: a linked population data study (2000–2012)

**DOI:** 10.1186/s12884-022-04736-6

**Published:** 2022-05-21

**Authors:** Hannah Grace Dahlen, Virginia Schmied, Cathrine Fowler, Lilian L. Peters, Simone Ormsby, Charlene Thornton

**Affiliations:** 1 School of Nursing and Midwifery, Western Sydney University, Locked Bag 1797, Penrith, NSW 2751 Australia; 2 School of Nursing and Midwifery, University of Technology, Broadway, Sydney, NSW 2007 Australia; 3 Department of Midwifery Science AVAG, Amsterdam UMC (location Vumc), Vrije Universiteit, Amsterdam Public Health Research Institute, Amsterdam, The Netherlands; 4 Department of General Practice and Elderly Care Medicine, University of Groningen, University Medical Center Groningen, Groningen, The Netherlands

**Keywords:** Residential parenting services, Early parenting, Perinatal mental health, Caesarean section

## Abstract

**Background:**

There is a tiered healthcare system in Australia to support maternal and child health, including, non-psychiatric day stay and residential parenting services (RPS) such as Tresillian and Karitane (in New South Wales [NSW]). RPS are unique to Australia, and currently there is limited information regarding the healthcare trajectory of women accessing RPS and if they are more likely to have admissions to other health facilities within the first-year post-birth. This study aimed to examine differences in hospital co-admissions for women and babies admitted to RPS in NSW in the year following birth compared to non-RPS admitted women.

**Methods:**

A linked population data study of all women giving birth in NSW 2000–2012. Statistical differences were calculated using chi-square and student t-tests.

**Results:**

Over the 12-year timeframe, 32,071 women and 33,035 babies were admitted to RPS, with 5191 of these women also having one or more hospital admissions (7607 admissions). The comparator group comprised of 99,242 women not admitted to RPS but having hospital admissions over the same timeframe (136,771 admissions). Statistically significant differences between cohorts were observed for the following parameters (*p* ≤ .001). Based upon calculated percentages, women who were admitted to RPS were more often older, Australian born, socially advantaged, private patients, and having their first baby. RPS admitted women also had more multiple births and labour and birth interventions (induction, instrumental birth, caesarean section, epidural, episiotomy). Their infants were also more often male and admitted to Special Care Nursery/Neonatal Intensive Care. Additionally, RPS admitted women had more admissions for mental health and behavioural disorders, which appeared to increase over time. There was no statistical difference between cohorts regarding the number of women admitted to a psychiatric facility; however, women attending RPS were more likely to have mood affective, or behavioural and personality disorder diagnoses.

**Conclusion:**

Women accessing RPS in the year post-birth were more socially advantaged, had higher birth intervention and more co-admissions and treatment for mental health disorders than those not accessing RPS. More research is needed into the impact of birth intervention and mental health issues on subsequent parenting difficulties.

## Background

In Australia there is a tiered system of health services (primary, secondary, tertiary) to support parents navigating early parenting difficulties. These include, non-psychiatric day stay and residential parenting services (RPS) such as Tresillian and Karitane (in NSW). In most instances RPS are led by nurses with support from multidisciplinary teams including paediatricians, psychologists and counsellors. Women and babies identified to be in need are referred to RPS by universal child and family health services or general practitioners, paediatricians, midwives, and allied health professionals. Some centres in addition accept women who self-refer [Dahlen et al., 2022, manuscript under review]. All Australian states and territories, except for Tasmania and the Northern Territory, have at least one RPS. In NSW (Australia’s most populace state), 7453 women accessed RPS over the 2018–2019 timeframe, of which 5793 attended Tresillian (three units), and 1660, Karitane (two centres) [1, 2]; representing 7.8% of the NSW birthing population [3]. During 2020-2021, Tresillian RPS admissions decreased slightly to 5699 (personal communication) as services remained open during the COVID-19 lockdowns to further assist parents cut off from their typical social support networks, however some hesitancy to attend facilities was noted. In the same time period, Karitane also reported lower numbers (1224, personal communication), as the service was transitioned to a virtual residential unit [4]. The range of RPS services provided include support with feeding, settling, infant caretaking skills and adjustment to parenting [5, 6]. More recently, an increased focus has also been placed upon psychosocial assessment and intervention [7], parental self-efficacy and parent-infant relationship development [8].

Poor mental health in the perinatal period is a global problem [9]. Recent Australian estimates for the occurrence of perinatal depression and anxiety are reported to be 20% for mothers and 10% for fathers [10]. Detrimental consequences include risk of self-harm, poor physical health, breakdown in relationships, social withdrawal, unhappiness in the parental role and, for some, lowered capacity to care for their infant [9, 11–14]. A small proportion of the perinatal population experience a severe mental health disorder requiring admission to an inpatient psychiatric unit or mother-baby psychiatric unit. Christl et al. (2015) report that the most common primary diagnosis for women admitted to mother-baby psychiatric inpatient units is a major depressive disorder, with more severe illness (schizophrenia and other psychotic disorders) accounting for between 20 and 40% of admissions [15]. While rates vary, many women admitted to hospital for mental health conditions have a history of mental illness [16]. This is a crucial concern because research shows that the early years of life strongly influence infant and childhood development [17, 18]. Poorer cognitive functioning; language impairment; and physical, psychosocial, emotional and behavioural problems have been identified in infants of women with perinatal mental health concerns [19–24].

Compromised physical health has also been associated with poorer mental health outcomes, with mothers experiencing five or more physical issues being identified in one Australian study, as six times more likely to report concurrent depressive symptoms at three months postpartum [25]. Typical complaints include tiredness, back pain, breast issues, perineal discomfort and urinary incontinence [25, 26]. Perineal trauma [27] and caesarean section [25] are additional sources of pain and discomfort for women during this time; all of which can contribute to or exacerbate postnatal mental health concerns [25, 26]. Physical pain can reportedly be more intensely felt when birth is experienced as traumatic [28].

While some of these services have been utilised for over 100 years, there has been limited research examining the characteristics of parents utilising RPS [29–31]. Findings from our recent population-based linkage study however identified that women accessing RPS services within the first-year post-birth were more socially advantaged, and had higher rates of birth intervention. In addition, over the ten-year timeframe examined, there was a significant increase in the proportion of women admitted to RPS that had instrumental births [32]. Other studies have similarly identified that women who have birth interventions are more likely to be admitted to RPS [33, 34], and that significant levels of birth trauma are often associated with labour and birth complexity as well as the use of interventions [35, 36]. It follows that these outcomes contribute towards parenting stress and impact upon postpartum depression [35].

To date, no studies have examined whether the health care trajectories of women admitted to RPS that also have hospital co-admissions within the 12 months following birth differ from non-RPS admitted women having hospital admissions during the same timeframe. We therefore wished to explore whether co-admissions of women and infants admitted to RPS in NSW during the period from 2000 to 2012 significantly differed from those observed in women not admitted to RPS but also having a hospital admission within 12 months following birth.

## Methods

Ethical approval was obtained from the NSW Population and Health Services Research Ethics Committee, HREC/10/CIPHS/96.

### Data sources

Data were obtained over the timeframe from January 1st 2000 to December 31st 2011 from the NSW Centre for Health Record Linkage (CHeReL) for which the following datasets were selected and linked: 1. Pregnancy and birth data provided by the NSW Ministry of Health from the Perinatal Data Collection (PDC), which contains statistics on all births of greater than 400 g birth weight and/or 20 completed weeks gestation, amounting to approximately one third of all Australian births annually. 2. The Admitted Patient Data Collection (APDC), which records all admitted patient services provided by NSW Public Hospitals, Public Psychiatric Hospitals, Public Multi-Purpose Services, Private Hospitals, and Private Day Procedures Centres. 3. Mortality data from both the APDC (discharge status) and the NSW Registry of Births, Deaths and Marriages (RBDM, death data). Bureau of Statistics Socio-Economic Indexes for Areas (SEIFA) codes were then applied to the cohorts to establish socio-economic (Index of Relative Socioeconomic Advantage and Disadvantage [IRSAD]) and education status (Index of Education and Occupation [IEO]) [37]. Probabilistic data linkage techniques were utilised for data linkage, whereby probabilistic record linkage software assigned a ‘linkage weight’ to pairs of records. For example, records that match perfectly or nearly perfectly on first name, surname, date of birth and address have a high linkage weight indicative of a probable match, whereas records matching only on date of birth have a low linkage weight (possible mismatch). This technique has been shown to have a false positive rate of 0.3% [38]. De-identified datasets were provided for analysis.

### Subjects

Two cohorts were created: Cohort 1 included all women who had an admission to a RPS for early parenting difficulties including sleep, feeding and settling issues; as well as a further admission/s to a NSW hospital for any medical reason/procedure within the 12-month period following birth. These women were identified utilising Tresillian and Karitane codes. Cohort 2 was comprised of all women who had a hospital admission for any medical reason/procedure in the same time period but not a RPS admission. During the 12-year data collection timeframe, it is possible that women having multiple events were included more than once in the dataset, and that the trajectories of their subsequent births may have differed in each case. By making comparisons between cohort 1 and 2 within the 12-month period post birth only, we have ensured that the same woman is not doubly included in these specific analyses. An individual woman may however be included in additional cohort comparisons in other 12-month post-birth timeframes. Admissions to psychiatric facilities were also obtained from the APDC. These sub-cohorts included any admissions within the time frame of the study and were not limited to the 12-month period following birth, due to the small sample size that would have obtained. In this case, it is possible that an individual women may have been included more than once in either cohort for the analyses. Data from subsets of these cohorts has also been provided in our previous study [32].

### Outcomes

Maternal antenatal, birth and neonatal data were extracted, including variables such as mode of birth, birth weight, parity, the presence of maternal hypertension or diabetes, and perinatal mortality. Gestation was recorded at birth (delivery gestation), as well as that obtained from the first antenatal appointment (booking gestation), which typically is calculated from the woman’s menstrual history, and/or the size estimate at the routine 12–13-week scan. International Classification of Diseases (ICD-10-AM) codes [39] and the Commonwealth Medicare Benefits Schedule procedure codes [40] were used for grouping of admission data. Diagnoses were obtained from both the primary coding and additional coding combined.

### Data analysis

The analyses between the two cohorts were conducted utilising contingency tables. Statistical differences were calculated with chi-square tests. Continuous variables were compared with student t-tests when normally distributed. Taking into account the size of the cohort and the number of analyses undertaken, results were considered statistically significant at the level *p* ≤ 0.01. Differences between cohorts were reported according to calculated percentages. Analysis was undertaken with IBM SPSS v.23®.

## Results

In cohort 1, 32,071 women and 33,035 babies were admitted to RPS with 5191 (16.2%) of these women also having a hospital admission (7607 admissions). In cohort 2, 99,242 women not admitted to RPS had hospital admissions (136,771 admissions). These admissions occurred in 382 facilities, 51.3% of which were public facilities. With respect to calculated percentages, it is notable that between cohort percentage differences are minimal in some categories, yet significant due to comparisons being made to the much larger sample size of cohort 2.

Comparisons between cohorts identified a number of demographic parameters for which differences were statistically significant (*p* ≤ 0.001, Table 1). Women in cohort 1 were older, and more often Australian born, socially advantaged, better educated, classified as a private hospital patient, and having their first baby, than women in cohort 2.

**Table 1:** Demographic profile of cohort 1 compared to cohort 2

Demographic	Residential (Cohort 1) *n* = 5191	Non-residential (Cohort 2) *n* = 99,242	*P* value
Age	30.5 (5.7)	29.4 (6.1)	**≤0.001**
Private patient	10.4%	7.07%	**≤0.001**
Australian born	80.6%	75.3%	**≤0.001**
Nulliparous	83.2%	66.2%	**≤0.001**
SEIFA^a^(> 6th decile - Index of Socio-economic Advantage & Disadvantage [IRSAD])	67.5%	50.0%	**≤0.001**
SEIFA^a^ (>6th decile - Index of Education & Occupation [IEO])	61.8%	51.2%	**≤0.001**

- Data are mean ± (SD) or (%) of cases within groups

^a^SEIFA deciles range from 1 (lowest) to 10 (highest), with >6th representing areas of above average socio-economic advantage/higher education & skilled occupation status

Statistically significant differences were also identified with respect to maternal perinatal and neonatal outcomes (*p* ≤ 0.001, Table 2). When compared to women in cohort 2, women in cohort 1 more often had a multiple birth, induction of labour, episiotomy, instrumental birth, caesarean section, and an epidural or some other type of pain relief. In addition, they more frequently had a male infant and a baby admitted to Special Care Nursery/Neonatal Intensive Care (SCN/NICU). However, women in cohort 1 were less likely to smoke and have a baby die.

**Table 2:** Maternal perinatal and neonatal birth outcomes

Variable	Residential women (Cohort 1) *n* = 5191	Non-residential women (Cohort 2) *n* = 99,242	*P* value
Booking gestation	9.8 (5.5)	10.6 (6.6)	0.03
Plurality (singleton)	94.4%	97.8%	**≤0.001**
Hypertension	4.9%	4.7%	0.42
Diabetes	5.4%	5.3%	0.97
Smoking during pregnancy	9.8%	18.6%	**≤0.001**
Delivery gestation	38.5 (2.86)	38.6 (2.99)	0.44
Induction	31.4%	29.3%	**≤0.001**
Delivery type			
- Normal vaginal	46.0%	53.6%	**≤0.001**
- Forceps vaginal	7.1%	5.4%	**≤0.001**
- Ventouse vaginal	11.9%	8.9%	**≤0.001**
- Vaginal breech	1.2%	1.1%	0.32
- Caesarean section	33.8%	31.0%	**≤0.001**
- Elective	46.9%	46.2%	0.60
- Emergency	53.1%	53.8%	0.60
Pain relief (% women who laboured)			
- None	8.2%	12.3%	**≤0.001**
- Epidural	33.6%	22.9%	**≤0.001**
Episiotomy (% of vaginal births)	26.6%	20.0%	**≤0.001**
Male sex	55.0%	51.8%	**≤0.001**
Birth weight	3261.2 (700.85)	3269.2 (713.70)	0.42
Apgar< 7	3.6%	4.4%	**0.004**
Admitted SCN/NICU	24.6%	22.2%	**≤0.001**
Resuscitation any form	47.9%	46.1%	0.01
Perinatal Mortality Rate (Within 42 days of birth)	17.0/1000 births	24.4/1000 births	**≤0.001**
Maternal Mortality rate (Within 12 months of birth)	5.8/10000 births	11.0/10000 births	0.40

- Data are mean ± (SD), (%) of cases within groups or specified fractions

In terms of hospital admissions, statistically significant differences between cohorts were again identified (*p* ≤ 0.001, Table 3). When compared to women in cohort 2, women in cohort 1 more frequently had an admission during the year following birth for mental and behavioural disorders, malignant neoplasms, and diseases of the musculoskeletal system and connective tissues (ICD-10-AM). Women in cohort 1 also less often had another baby within the 12-month period following the index pregnancy.

**Table 3:** Diagnostic codes (ICD-10-AM) recorded for maternal readmission in the 12-month period following delivery

ICD-10-AM Grouping Name		Codes	Cohort 1 *n* = 5191	Cohort 2 *n* = 99,242	*P* value
Certain infectious & parasitic diseases		A00-B99	2.34%	2.72%	0.02
Neoplasms	Malignant neoplasms	C00-C96	1.58%	1.33%	≤**0.001**
	In situ neoplasms	D00-D09	0.31%	0.47%	**0.008**
	Benign neoplasms	D10-D36	1.03%	0.89%	0.013
	Neoplasms of uncertain or unknown behaviour	D37-D48	1.27%	1.22%	0.2
Diseases of the blood & certain blood forming organs & certain disorders involving the immune mechanism		D50-D89	0.68%	0.84%	0.07
Endocrine, nutritional & metabolic diseases		E00-E89	1.26%	1.40%	0.36
Mental & behavioural disorders		F00-F99	8.43%	4.18%	≤**0.001**
Diseases of the nervous system		G00-G99	0.97%	0.93%	0.3
Diseases of the eye & adnexa		H00-H59	0.27%	0.30%	0.85
Diseases of the ear & mastoid process		H60-H95	0.09%	0.17%	0.03
Diseases of the circulatory process		I00-I99	1.59%	1.65%	0.94
Diseases of the respiratory system		J00-J99	1.40%	1.61%	0.14
Diseases of the digestive system		K00-K93	7.82%	8.07%	0.63
Diseases of the skin & subcutaneous tissue		L00-L99	0.93%	1.01%	0.68
Diseases of the musculoskeletal system & connective tissue		M00-M99	1.63%	1.38%	≤**0.001**
Diseases of the genito-urinary system		N00-N99	6.30%	6.33%	0.15
Pregnancy, childbirth & the puerperium		O00-O99	19.38%	20.87%	≤**0.001**
Congenital malformations & chromosomal abnormalities		Q00-Q99	0.30%	0.27%	0.32
Symptoms, signs & abnormal findings, clinical & laboratory findings not elsewhere classified		R00-R99	4.65%	4.56%	0.06
Injury, poisoning & certain other consequences of external causes		S00-T98	2.47%	2.74%	0.22
External cause of morbidity & mortality		U50-Y98	6.44%	6.51%	0.22

-ICD-10-AM - International Statistical Classification of Diseases & Related Health Problems, Tenth Revision, Australian Modification (ICD-10-AM) used to classify diseases & other health problems

When comparing the 10 most commonly occurring procedures provided to the cohorts (Table 4), we again found statistically significant differences between the cohorts (*p* ≤ 0.001). Women in cohort 1 less frequently had anaesthesia, and major surgery (of all body systems apart from gynaecological), however more often received allied health intervention, chemotherapy and psychological therapy when compared to women in cohort 2.

**Table 4:** Ten most commonly occurring procedures conducted on admitted women in the 12-month period following delivery

Procedure classification	Cohort 1 *n* = 5191*	Cohort 2 *n* = 99242*	*P* value
Anaesthesia	20.3%	21.5%	≤**0.001**
Major surgical (all body systems except gynaecological)	9.53%	10.44%	≤**0.001**
Diagnostic procedures	7.86%	7.84%	0.01
Allied health intervention	7.8%	6.7%	≤**0.001**
Psychological therapy	4.54%	1.15%	≤**0.001**
Minor surgical (all body systems except gynaecological)	1.97%	2.13%	0.18
Dental	1.8%	1.5%	**0.008**
Transfusion	0.5%	0.7%	**0.004**
Cosmetic procedures	0.30%	0.28%	0.78
Chemotherapy	0.2%	0.1%	≤**0.001**

- Data are (%) within code classification; * 14,699 procedures were conducted for cohort 1, & 264,849 for cohort 2

Examination of the incidence of gynaecological and obstetric procedures conducted similarly identified statistically significant differences between the cohorts (*p* ≤ 0.001, Table 5). Women in cohort 1 more often experienced IVF procedures (*p* = 0.004), whereas less contraception/sterilisations, termination of pregnancies, and obstetric interventions (insertion of suture/diagnostic procedure/postpartum dilatation & curettage) when compared to cohort 2.

**Table 5:** Gynaecological and obstetric procedures conducted on women in the 12-month period following delivery

Classification	Procedure	Cohort 1 *n* = 5191*	Cohort 2 *n* = 99,242*	*P* value
Minor surgical		0.28%	0.36%	0.14
Major surgical	Oophorectomy/salpingectomy/ectopic pregnancy removal	0.22%	0.34%	0.03
	Hysterectomy	0.10%	0.12%	0.70
	Diagnostic procedure	0.40%	0.51%	0.08
	Myomectomy/cystectomy	0.28%	0.31%	0.58
	LLETZ procedure/ovarian/uterine/pelvic/vaginal lesions	1.18%	1.24%	0.50
	Repair of rectal prolapse/rectocele/anorectalplasty/anoplasty/sphincterplasty/anal repair/anal fissure/thrombus/abscess/anal examination/insertion of anal stimulator/repair of anal fistula	0.33%	0.32%	0.84
	Vagina/uterus/pelvic floor/urethral/cervix repair/vaginal orifice surgery/perineal haematoma/perineal repair	0.77%	0.68%	0.24
	Caesarean section wound haematoma	0.03%	0.09%	0.05
	Cystocele/enterocele/repair for incontinence	0.14%	0.11%	0.33
	Vulvoplasty/cliteroplasty	0.12%	0.08%	0.07
	Other uterine procedures	0.06%	0.07%	0.49
	Other female reproductive procedures	0.35%	0.39%	0.48
Contraception/sterilisation		0.69%	1.61%	≤**0.001**
IVF		1.86%	1.56%	**0.004**
Termination/dilatation and curettage		4.00%	5.13%	≤**0.001**
Obstetric	Insertion of suture/diagnostic procedure/postpartum dilatation & curettage	3.31%	4.13%	≤**0.001**

- Data are (%) within code classification; * 14,699 procedures were conducted for cohort 1, & 264,849 for cohort 2

When further examination of admission codes was conducted for maternal mental and behavioural disorders (Table 6), we again found statistically significant differences between groups (*p* ≤ 0.001). We found mood affective disorders; neurotic, stress-related and somatoform disorders; behavioural syndromes associated with psychological disturbances and physical factors; and personality and behaviour disorders more often occurred in cohort 1 than in cohort 2. This was most striking for mood affected disorders (5.4% vs 2.4%), behavioural syndromes (4.3% vs 1.2%), and neurotic, stress-related and somatoform disorders (4.5% vs 1.8%).

**Table 6:** Mental and behavioural disorders (F00-F99) compared between cohort 1 and 2

ICD-10 Mental & behavioural disorders Grouping Name	Codes	Cohort 1	Cohort 2	*P* value
Mental & behavioural issues due to use of alcohol & drugs	F10-F19	2.8%	2.7%	0.11
Schizophrenia, schizotypal & delusional disorders	F20-F29	0.7%	0.8%	0.9
Mood affective disorders (mania, bipolar affective disorder, depressive disorder, mood disorders)	F30-F39	5.4%	2.4%	**≤0.001**
Neurotic, stress-related & somatoform disorders (phobias, anxiety disorders, somatoform disorders, neurotic disorders)	F40-F48	4.5%	1.8%	**< 0.001**
Behavioural syndromes associated with psychological disturbances & physical factors (nonorganic sleep disorders, sexual dysfunction, mental & behavioural disorders associated with the puerperium, harmful use of non-dependence substances)	F50-F59	4.3%	1.2%	**≤0.001**
Disorders of adult personality & behaviour	F60-F69	1.7%	0.7%	**≤0.001**

- ICD-10 International Statistical Classification of Diseases & Related Health Problems, Tenth Revision, Chapter V: Mental & behavioural disorders - F00-F99

- Data are (%) of cases within groups

**Fig. 1:**
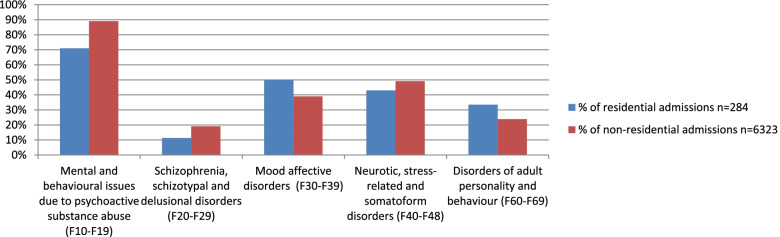
Five most common psychiatric codes assigned to women admitted to psychiatric facilities between women who were also admitted to RPS and those who were not

**Fig. 2:**
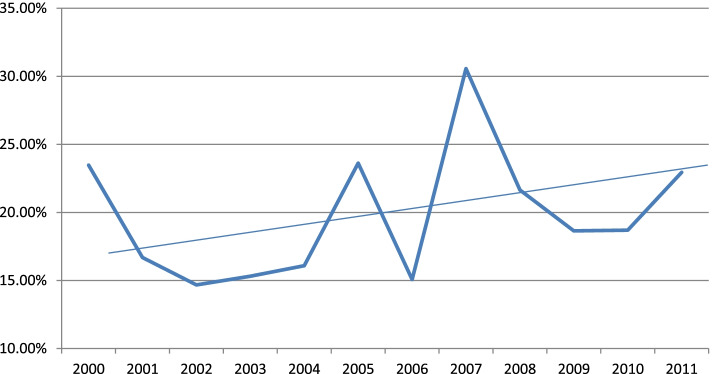
Psychiatric ICD-10-AM codes as a percentage of all coding of admissions to residential units expressed over time -ICD-10-AM - International Statistical Classification of Diseases & Related Health Problems, Tenth Revision, Australian Modification (ICD-10-AM) used to classify diseases & other health problems

With respect to any admission to a psychiatric facility, there were no statistically significant differences between cohort 1 and 2 (1.03% vs 1.09%, *p* = 0.44), however statistically significant differences were noted in regard to diagnosis types (*p* < 0.001, Table 7). Women admitted to RPS more often received a diagnosis of a mood affective disorder (50.0% vs 39.0%) and/or a disorder of adult personality and behaviour (33.5% vs 23.9%) when compared to women not admitted to RPS. However, women in cohort 1 less often had mental and behavioural issues arising as a consequence of psychoactive substance abuse (71.0% vs 89.0%) and schizophrenia, schizotypal or delusional disorders (11.3% vs 19.1%). These differences are represented in Fig. 1, along with the findings for neurotic, stress-related and somatoform disorders (*p* = 0.05). It was also interesting to note that over the 12-year study period, it was observed that there was an overall 7% increase in the number of women in cohort 1 having a psychiatric diagnosis noted on their RPS admissions (Fig. 2).

**Table 7:** ICD-10-AM coding assigned to women admitted to psychiatric facilities comparing RPS and non-RPS cohorts

ICD-10-AM coding description	Codes	Residential admissions *n* = 284	Non-residential admissions *n* = 6323	*P* value
Organic, including symptomatic, mental disorders	F00-F09	0.4%	0.8%	0.02
Mental & behavioural issues due to psychoactive substance abuse	F10-F19	71.0%	89.0%	**< 0.001**
Schizophrenia, schizotypal & delusional disorders	F20-F29	11.3%	19.1%	**0.001**
Mood affective disorders	F30-F39	50.0%	39.0%	**< 0.001**
Neurotic, stress-related & somatoform disorders	F40-F48	43.0%	49.1%	0.05
Behavioural syndromes associated with psychological disturbances & physical factors	F50-F59	5.6%	4.1%	0.26
Disorders of adult personality & behaviour	F60-F69	33.5%	23.9%	**< 0.001**
Mental retardation	F70-F79	1.4%	1.1%	0.85
Disorders of psychological development	F80-F89	0.7%	0.5%	0.74
Behavioural & emotional disorders with onset usually occurring in childhood & adolescence	F90-F98	2.8%	1.9%	0.38
Unspecified mental disorder	F99	0.0%	0.0%	

- Data are (%) of cases within groups

## Discussion

This is the largest study to date examining the characteristics of women and babies admitted to RPS in NSW who also had a co-admission in the year following birth. The comparison of this cohort to women not admitted to RPS but also having a hospital admission during the same time period is unique to this study. We found that the women who attended RPS in NSW and had a co-admission in the year following birth were slightly older, and more likely to be born in Australia, a private patient when admitted, socially advantaged (higher SEIFA index) and having their first baby. We also identified that these women had higher rates of birth intervention and increased incidences of co-admissions and treatment for mental health disorders.

### Socio demographics

Even though these women were identified as socially and economically advantaged they may still have lacked the necessary social supports to develop confidence in their ability to parent while adjusting to the parenting role and changes in lifestyle that are required with motherhood [41]. Lack of functional support from family, friends or community has frequently been indicated as an issue for mothers admitted to RPS [33, 42]. This type of support is consistent with that provided to women admitted to RPS and includes informational, instrumental, emotional and appraisal support [43], which has been demonstrated to positively influence parenting self-efficacy and lessen the likelihood of experiencing postnatal depression symptoms [43].

### Birth intervention

The significantly increased likelihood of experiencing birth intervention and a caesarean section in the RPS group may have been due to increased complexity in these pregnancies, yet there was no higher incidence of diabetes or hypertension; and smoking was in fact significantly lower. Rather, these factors have been shown to be more related to women who are socially advantaged and receiving private obstetric care in Australia [44], despite evidence of increased morbidity for these babies (especially in regard to scalp trauma) and no perinatal advantage [45, 46]. There is also increasing evidence that women who experience higher rates of medical intervention during labour and birth are more likely to suffer from birth trauma [35] and postnatal depression and have babies affected by gastro-oesophageal reflux and settling and sleeping disorders [34]. It was of interest to note that the higher rates of birth intervention in the RPS group was significantly associated with increased SCN/NICU admissions, and lower Apgar for babies, but lower mortality rates, most likely due to their social advantage.

### Mental health

We found significantly more women who had been admitted to RPS had hospital admissions for mood, stress-related, personality and behavioural disorders/syndromes, as well as inpatient admissions to psychiatric facilities. These results are consistent with previous research that demonstrated significant levels of depression and anxiety amongst women admitted to RPS [47–49] [Dahlen et al., 2022, manuscript under review].

While women experiencing significant mental illnesses may have developed these prior to pregnancy or birth [50, 51], the perinatal period is recognised as a time of particular vulnerability for onset and/or exacerbation of mental health conditions [52]. Consequently, routine antenatal screening for mental health disorders has been introduced into Australian public hospitals [53], however screening appears not to be as widely implemented in private settings [53, 54]. Further education of potential postnatal difficulties may also be beneficial for mothers at this time, as approximately one in five Australian women with a full-term infant have symptoms of a mental health condition or meets the Diagnostic and Statistical Manual of Mental Disorders (DSM-V) criteria for perinatal mental illness in the first year following birth [55–57]. It has also been estimated that two thirds of postnatal women with depression or anxiety were symptomatic during pregnancy, with migrant women more likely to be affected with postnatal depression (24–42% compared to 10–15%) [56, 58, 59].

According to a recent NSW study, severe psychiatric disorders resulting in hospital admissions of primiparous mothers within the first-year post-birth have increased significantly over the time period from 2001 to 2010, with rates of admission at the latter end of this period occurring in just over 2% of women [60]. We also observed a 7% increase in the proportion of women having a psychiatric diagnosis recorded on their RPS admission between 2000 and 2012. The most common reasons for these admissions were mood affective, and personality and behavioural disorders.

We recently conducted an integrated review of 40 studies assessing RPS within Australia, in which we confirmed mental health issues arising as a consequence of or exacerbated by parenting difficulties are common and associated with disturbed infant feeding, behaviour, sleep and settling [Dahlen et al., 2022 manuscript under review]. We also found these issues were coupled with negative reproductive experiences; compromised physical health; stress, lack of support and/or other psychosocial risk factors; fatigue; low self-efficacy; and poor relationship quality amongst parents; and thereby increasing life complexity [Dahlen et al., 2022 manuscript under review]. Further compounding of these factors can also occur as a consequence of fatigue [61] and mental illness decreasing maternal sensitivity and the ability of parents to provide their infants with the necessary consistent and sensitive care that is required [47, 62].

While RPS predominately focus on the care of the infant and the developing parent-infant relationship, it also enables mothers (and in some cases fathers) to receive mental health assessment and referral to specialist mental health professionals and services. The initial perceived focus on the infant may facilitate the subsequent seeking of parenting and mental health assistance by reducing the fear of consequence and stigma associated with disclosing mental illness and parenting difficulties [63]. These ideas are consistent with the Australian government’s emphasis on mental health and social inclusion; early intervention; service access and recovery; continuity of care; and management coordination. While much work has been done in the area of mental health, more is needed to ensure all parents, regardless of their socioeconomic status, have access to appropriate support and treatment services.

### Physical health

It would appear that there is an increasing proportion of women becoming pregnant with existing chronic health issues, such as diabetes, obesity and cardiac disease [64, 65]. The incidence of physical health problems arising after birth is also on the rise and reportedly due to a range of complex factors, inclusive of social issues, the increasing administration of birth interventions, and a bi-directional relationship with maternal mental disorders [25, 26]. Caesarean section rates for example are increasing [66, 67], as are the associated morbidities of haemorrhage requiring a hysterectomy, uterine rupture, major puerperal infection, venous thromboembolism, cardiac arrest, renal failure, obstetric shock, and in the longer-term, pelvic adhesions and bowel obstructions [67]. Severe perineal trauma resulting in pain, incontinence and painful urination is also more common and not surprisingly associated more with instrumental birth.

### Limitations

This paper examines admissions to hospitals and residential parenting facilities only and therefore is limited by the fact that visits to general practitioners, community based and outpatient facilities are not included in the linked dataset.

It is not possible to draw a direct link between higher rates of intervention during the birth and increased likelihood of having an admission to a RPS, as other factors such as having higher socio-economic and education levels that comes with social advantage could lead to an increased uptake of services, especially as these women are also more likely to receive private obstetric care which is associated with increased intervention. The variations in the psychiatric diagnoses of the women accessing RPS may also be an association that is not directly linked and therefore research is needed to further explore this possibility. As the data set was collected over the period from January 1st 2000 to December 31st 2011, potential impacts of the COVID-19 pandemic are not included in this study. We however anticipate that the global pandemic would have further detrimentally impacted upon maternal postpartum mental health. This will be explored in ongoing work.

## Conclusion

This study is unique in that it examines a complete cohort of women who were admitted to RPS in NSW that also had co-admissions to hospitals within the first-year post-birth and compares them to women who were not admitted to RPS but had a hospital admission during the same time period. Findings demonstrated that women who accessed RPS in the first year after birth were more often first-time mothers, socially advantaged, having higher birth intervention and more co-admissions and treatment for mental health disorders than those who did not access RPS. More research is needed to explore the impact of birth intervention on mental health issues and subsequent parenting difficulties.

## Data Availability

Data is available from the NSW CHeReL. Further details can be obtained from the corresponding author.

## Abbreviations

APDC: Admitted Patient Data Collection
CHeReL: Centre for Health Record Linkage
DSM: Diagnostic and Statistical Manual of Mental Disorders
HREC: Health Services Research Ethics Committee
ICD: International Classification of Diseases
IVF: In-vitro Fertilisation
NICU: Neonatal Intensive Care Unit
NSW: New South Wales
PDC: Perinatal Data Collection
RBDM: Registry of Births, Deaths and Marriages
RPS: Residential Parenting Services
SCN: Special Care Nursery
SEIFA: Socio-Economic Indexes for Areas
